# Some Aspects of Medical Science

**Published:** 1894-07-14

**Authors:** 


### Some Aspects of Medical Science.

In two previous papers we have discussed the relative sexual differences in acuteness and precision of sensory perceptions, in the cases of sensitiveness to pain and the sense of smell. We propose now in this paper to consider the sense of taste as exhibited in the two sexes.

These sexual differences in the sense of taste, like those in the sense of smell, seem to have been first accurately measured by Professors Bailey and Nickols, we are reminded by Mr. Havelock Ellis, who gives us an abstract of their work undertaken in this direction

The two eminent American scientists commenced their operations with a series of strong solutions for the different classes of sapid substances: for bitter, quinine was selected (1 part in 10,000 parts of water); for sweet, cane sugar (1 part in 10 of water); for acid, sulphuric acid (1 in 100); for alkaline, sodium bicarbonate (1 in 10); for saline, common salt (1 in 100). By successive dilutions each of these solutions became the strongest of a series, each member of which was one half the strength of the preceding one. The bottles containing the solutions were then placed without any regard to order, and the person experimented upon was required to taste and separate them in their proper grouping. They were so diluted that in each series the last solution was quite unrecognisable. The following table will give the result of the test as the professors applied it; they examined 128 persons, of whom 82 were male, 46 female:—

| | Male observers detected | Female observers detected |
|---|---|---|
| Quinine. | One part in 392,000 | One part in 456,000 |
| Sugar ... | „ „ 199 | „ „ 204 |
| Acid ... | „ „ 2,080 | „ „ 3,280 |
| Alkali... | „ „ 98 | „ „ 126 |
| Salt ... | „ „ 2,240 | „ „ 1,980 |

The result of this particular investigation proves that the sense of taste is certainly of a more delicate character in women than in men, in spite of the fact that men have the monopoly of the higher walks of the culinary art. The case of salt proved, however, exceptional, as we may notice. Speaking of their own experiments the professors remark that they had found precisely a similar difference "in an earlier and independent set of experiments, which agreed in every essential particular with the results of the present test; we do not regard it as an accidental difference, or as likely to disappear in more extended investigations."

Mr. Ellis tells us that wide individual differences presented themselves, as much as in the ratio of one to three, variations which were not explainable as the results of education, because in many cases men who had had immense experience in the handling of drugs were surpassed by women who had had none.

Later on, and quite independently of the above work, this question was taken up in Turin by Dr. Ottolenghi,* whose name we have before had occasion to mention in connection with previous research. The results, however, seem to have been not quite in accord with the American conclusions. In Turin 190 persons, *i.e.*, 60 male congenital criminals, 20 male occasional criminals, 20 normal males of the lowest social class, 50 students and professional men, 20 criminal women and 20 normal women were experimented with; the greater part of the entire number of this varied gathering were of healthy and robust constitution, and ranging in ages from 20 to 50; the doctors experimental tests being with bitter, sweet, and salt sensations.

For the first he selected sulphate of strychnine, and discovered 12 per cent. of his normal persons perceived 1 part in 800,000. Setting out from this strength he made 11 graduated solutions, the strongest being 1 part in 50,000. As a sweet substance, in place of sugar, which is not very divisable, he used saccharine, making 11 gradations between 1 in 100,000 parts (which could be tasted by 25 per cent. of the normal men and 45 per cent. of the normal women, and 1 in 11,000; the 11 common salt solutions ranged from 1 in 500 to 3 in 100. Numerous precautions were taken, and Mr. Ellis tells us (to whose abbreviated form of the procedure we are in the present instance indebted) that these precautions were carried out in so thorough and efficient a manner that the mouths of those who thus lent themselves to the taste were rinsed with luke warm water!

Then, again, every one of the experiments was repeated twice, and control experiments with distilled water were made to avoid the disturbing influence of expectation and subjective sensations; such solutions being arranged in accord with the temperature of the air. In making the tests, the various solutions were squirted by a pipette on to the tongue, care being taken, we are assured, that the amount should always be precisely the same, this being half a c. cm.

Ottolenghi presents his results in tabular form, dividing the subject into three groups, which indicate delicate, middling, and obtuse sensations under each head of "bitter," "sweet," and "salt." The table is arranged in so orderly a manner, that one reads at a glance the percentage of individuals in relation to each solution. Speaking generally, as our English authority points out, the criminals, certainly the male criminals, showed small claim to belonging to a class characterised with any delicate sense of taste. But again, Dr. Ottolenghi asserts that in the case of the normal women who came under his experience, they ranked with professional men; 90 per cent. of these "normal" women were discovered to possess refined taste as regards salt, against 80 per cent. of the professional men, the difference in favour of the former being marked in the case of the weakest salt solution (90 per cent.) of the women to 40 per cent. of the men.

And what conclusion do the specified investigations which we have selected lead us to? We cannot help agreeing with an English authority on the subject, that men have a monopoly of the higher walks of culinary art. Women are rarely employed in such occupations as require specially delicate discrimination; they are rarely good wine-tasters—never tea-tasters. "Gourmet" has no feminine form in the vocabulary; and these, and many other reasons, combine to suggest an inferiority in respect to the sense of taste in women as compared with men. The above investigations, however, when we give them something more than a passing study, lead us to conclusions other than those we had previously formed.

* "Il Gusto nei Criminali in Rapporto coi Normali." Archivio di Psictriatria, Vol. x., Fasc iii.-iv. pp. 332-338.

